# Changes of collective orientation through a medical student’s anaesthesia simulation course – simulation-based training study with non-technical skills debriefing versus medical debriefing

**DOI:** 10.1186/s12909-019-1765-x

**Published:** 2019-09-05

**Authors:** Hendrik Eismann, Thomas Palmaers, Svetlozar Tsvetanov, Vera Hagemann, Markus Flentje

**Affiliations:** 1 Department of Anaesthesiology and Intensive Care Medicine, Hannover Medical School, Carl-Neuberg-Strasse 1, 30625 Hannover, Germany; 2 Faculty of Business Studies and Economics, University of Bremen, Enrique-Schmidt-Strasse 1, 28359 Bremen, Germany

**Keywords:** Collective orientation, TeamGAINS, Medical student, Learning, Simulation

## Abstract

**Background:**

Non-technical skills (NTS) are known to have a positive impact on quality of medical care. The team performance enhancing behaviour, as an example for NTS, is termed “Collective Orientation” (CO). In this study, we investigated the effect of a simulator-based anaesthesia training upon student’s CO in relation to medical and TeamGAINS (guided team self-correction, advocacy-inquiry and systemic-constructivist techniques) debriefing. We hypothesized (a) the scale collective orientation, as demonstrated in other team setting, is applicable to fourth year German medical students, (b) collective orientation increases by a four-hour anaesthesia simulation course, (c) the change in collective orientation can be influenced by type of debriefing.

**Method:**

All classes of an anaesthesia module (4th year medical students) were randomized into two groups. Students took part in a four-hour simulation course with team scenarios, supported by a simulated nurse. In group one the trainer focused on a debriefing on medical problems and in group two, a debriefing according to the specifications of the TeamGAINS concept was conducted. The primary outcome was the mean difference between the collective orientation measured (via questionnaires) immediately before (T1) and after (T2) training.

**Results:**

Cronbach’s alpha for all scales and measurement points was higher than 0.72. The scale “affiliation” decreases in the group medical debriefing MD = 0.1 (*p* = 0.008; r = 0.31) and was unchanged in the group TeamGAINS. “Dominance” increases in both groups. The values were MD = 0.19 (*p* = 0.003; r = 0.25) for medical debriefing and MD = 0.22 (*p* = 0.01; r = 0.40) for TeamGAINS debriefing.

**Conclusion:**

The collective orientation questionnaire can be applied to fourth year medical students. Simulation courses influence the attitude towards teamwork. The influence is negatively to the subscale “affiliation” by a “medical debriefing” and independently regardless of the nature of the debriefing for the subscale “dominance”. We recommend a debriefing for medical students using the TeamGAINS approach to clarify the connection between the individual performance and non-technical skills. Anaesthesia simulation courses have the potential being a part of a longitudinal education curriculum for teaching non-technical skills.

## Introduction

A successful medical treatment in the complex environment of health care systems is based on more competencies than individual medical knowledge. A high level of responsibility, an irreversibility of therapeutic decisions and significant time pressure are conditions of so called high risk organisations (HRO) [1]. For these organisations, teamwork has been identified as a factor for successfully dealing with critical incidents and avoiding accidents [2]. In interprofessional healthcare, teamwork and collaboration is recommended for good medical care [3]. For this reason, teamwork was taken to account during the creation of a guideline catalogue (German National Competence-Based Learning Objective) for the education of medical students in Germany. In section 1 a competence orientation for the physicians role as a team member [4] was described. With introduction of the catalogue in 2015, projects have been started to compare the contents with the reality of education in universities [5]. Many matching processes are still in progress. After further research, it will be interesting to see how the new teaching contents, such as team management, are prioritized by the students in competition for education time with the classic contents.

The anaesthesia setting is well suited to engage in interprofessional teamwork. Professional societies have done research in this area and defined “teamwork” as a core competency in the so-called non-technical skills in addition to “situation awareness”, “communication” and “task-management” [6]. The European Society of Anaesthesiology (ESA) published a recommendation as part of the Helsinki Declaration on Patient Safety for the utilization of periodic simulation-based training [7]. A high competence of anaesthesiologists in non-technical skills can be assumed. Non-technical skill trainings are often referred as “crisis resource management” courses. An important part of this trainings is the debriefing of the team after conducting simulation scenarios [8].

Individual parameters of success of a debriefing have not been conclusively clarified [9]. The TeamGAINS (guided team self-correction, advocacy-inquiry and systemic-constructivist techniques) approach for example claims to cover the areas surfacing, reflecting on and changing of the dynamics of team interactions [10].

Diskrell et al. defined teamwork as a propensity to work in a collective manner in team settings [11]. Calling this propensity “Collective Orientation” (CO), two dimensions became apparent during development of a measuring scale. The first is called “Affiliation” and describes the ability to work in a team, both in a goal-oriented manner and with a high regard for others. Low “Affiliation” is characterized by a preference to work on one’s own. The second factor was called “Dominance” and demonstrates a priority in having power and control over a cooperate working style. “Collective Orientation” is an attitude which effectively supports team processes [12, 13]. It can be positively changed by training of health care providers, if the participants believe it is important to their work [14]. The adapted German version of the questionnaire was developed by Hagemann [15]. Teamwork as an important factor of Non-technical-Skills [6]. Improvements in anaesthesiologists’ nontechnical competencies and teamwork via simulation-based training has previously been demonstrated to have a positive influence on the quality of patient care in the operation theatre [16, 17].

The aim of the study was to assess the impact of an anaesthesia simulation training upon fourth year medical student’s collective orientation in dependence of a debriefing with solely medical content or crisis resource management combined with medical content. The time frame of the existing course should not be extended, since these are defined framework conditions of the anaesthesia module. Our hypotheses were: (a) the scale collective orientation, as demonstrated in other team setting, is applicable to fourth year German medical students, (b) “Collective Orientation” increases by a four-hour anaesthesia simulation course, (c) the change in “Collective Orientation” can be influenced by type of debriefing.

## Methods

### Setting and population

**Fig. 1:**
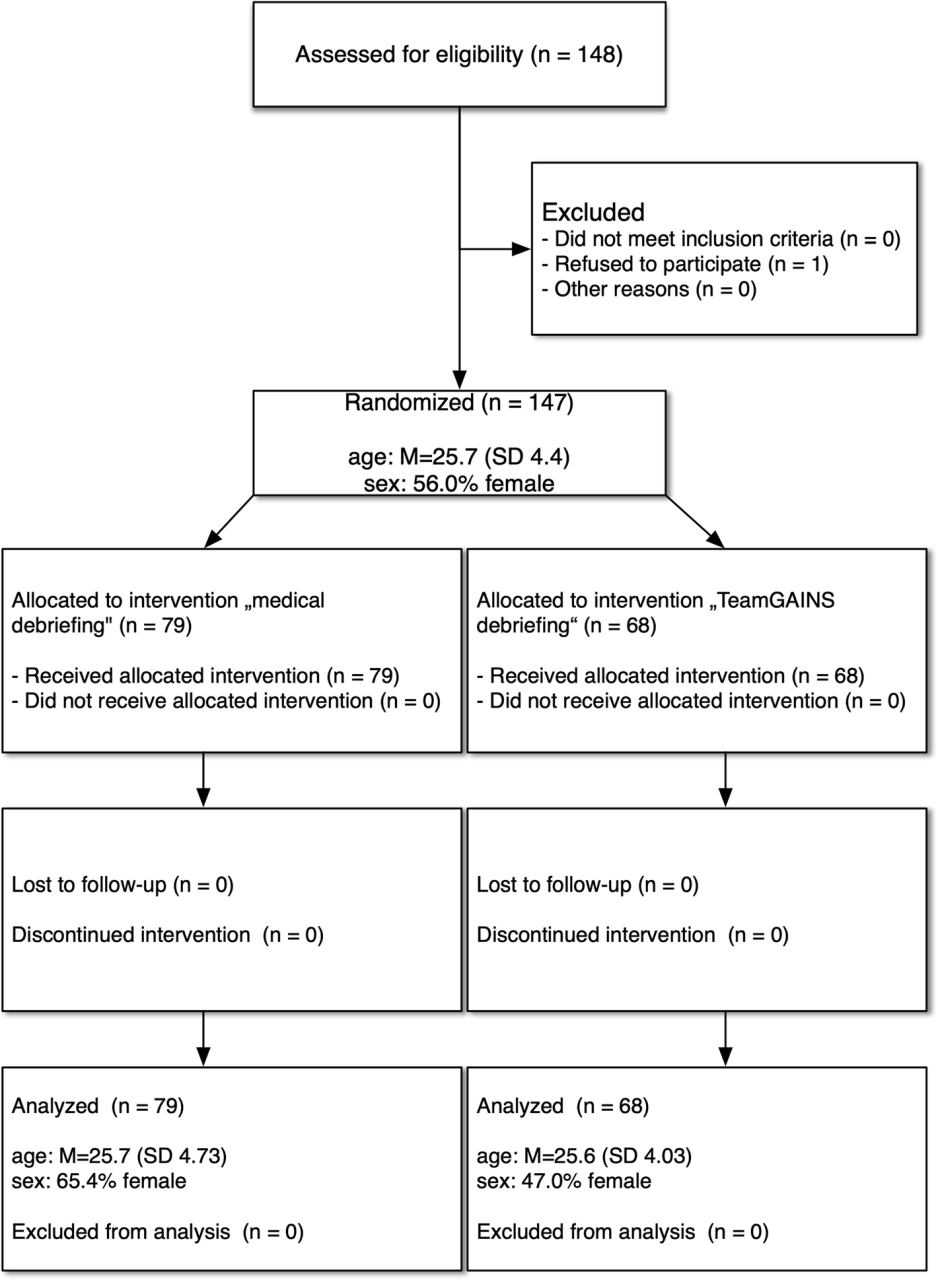
CONSORT flow diagram

This study has a between-group pre-post design using a survey methodology with participants enrolled in our medical student simulation program at Hannover Medical School simulation center. All students took part at the anesthesia module, which is offered and certified (written exam) in the fourth year of studying medicine. Before attending the course, the students had a four-hour seminar with a focus on perioperative management combined with an internship in the operation theatre. The lectures are held parallel to all simulation courses, so a difference in knowledge of the participants during the investigation period must be assumed. Participation in the lecture is optional. The process of the study is presented in Fig. 1.

### Training course

The medical student simulation program is a four-hour simulation course. It takes place at the simulation center of the department of anaesthesiology and intensive care medicine at Hannover Medical School. The course is designed for a group size up to twelve students. The assignment of the students to the individual course days is managed centrally by the office of the dean. The authors had no influence on the composition of the individual groups.

The scheduled course days were randomized into two groups using the online randomization tool at randomizer.org. Group one was labeled “medical debriefing”, group two was labeled “TeamGAINS” debriefing”. All steps of the TeamGAINS debriefing are shown in Table 1. Group one (“medical debriefing”) went through the steps one, two, three and six. All steps of the TeamGAINS concept were carried out to the second group (“TeamGAINS”), especially the CRM-related steps four and five. The students were unaware of the type of debriefing during the study and of their individual group allocation.

**Table 1:** The integrated approach for structured debriefing TeamGAINS as a six-step approach, developed from Guided team self-correction, Advocacy-Inquire, Systemic-constructivist the integrated approach for structured debriefing

Six Steps of the TeamGAINS debriefing approach [10]		
Step	Topics	Instuctor’s method and examples of communicaiton
1	Reactions	Narrative question (e.g. “How did you feel?”).
2	Debriefing the clinical part of the scenario, clarify questions, allow for understanding the appropriate clinical procedures	Narrative question, Advocay-inquiry (“What happened?”, “I saw you re-attempting to intubate using the laryngoscope three times”) Guided team self-correction (“What alternative device could you have used for intubation?”) Systemic constructivist approach: circular question (“What would he have recommended to the colleague?”)
3	Transfer from simulation to reality	Narrative question (“What aspects of this scenario are familiar to you from the real world?”)
4	Reintroduce the expert model, systematically discuss the behavioral skills and their relationship to clinical outcomes	Guided team self-correction: elicit reflection about positive behaviour (“Give me an example of a situation where you anticipated a potential complication.”) Systemic question (“Having anticipated the potential complication— how did this help you later on?”) Advocacy-inquiry -using the video (“Let’s talk about shared planning. During that situation, I saw you working very quietly together, and I was concerned whether each of you knew about each other’s plan for the next step. What was on your mind?“) Circular question (“How could it have been useful for him to know what you were about to do and what you needed?”) Observer-perspective, circular questions using the Reflecting Team (“What do you think she might have needed from him to speak up in that situation?”)
5	Summaries learning experience and finish debriefing	Inquiry (“Which of the CRM-principles do you consider most important after that situation?”) Circular question (“Overall, if inexperienced anaesthesia residents and nurses had watched you during the scenario, what could they have learned from you?”)
6	If required, improve clinical skills	Practice clinical skills, that were not optimally performed during the scenario (example: practice using the defibrillator)

All courses were conducted by two trainers (HE, MF), who received a two-day training in the TeamGAINS concept by the authors Kolbe and Grande [10]. All debriefings during the study phase were conducted by these two trainers. The course starts with a brief welcome and presentation of the course objectives. All students were informed of the goal to perform general anaesthesia in typical situations and to deal with common incidents of daily anaesthesia practice. The sequence of each scenario (briefing, scenario, audio-video-debriefing) was presented and a written consent for audio-video-recording was obtained. An oral agreement with the participants on compliance with debriefing rules (e.g. respect, hearing out, listen) was made. The objective of learning to master typical anesthesiologic scenarios, taking the skills of educational level into account, was presented. Group two (TeamGAINS debriefing) received an additional 10 min presentation of the ANTS (Anaesthetists’ Non-Technical Skills) framework [6]. TeamGAINS represents a structured debriefing model utilising different types of questions to scrutinize the individual mental models of decision making. We focussed mainly on non-technical skills, as decision making and team work are crucial aspects of this framework.

After an introduction to the workplace environment and the patient simulator (Laerdal SimMan 3G, Laerdal Medical, Norway), the participants took part in several simulations e.g. “elective anaesthetic induction”, “rapid sequence induction”, “allergic reaction”. In each scenario, two students cooperated with a “nurse”, simulated by an instructor.

The simulated nurse performed all tasks within their scope of practice (e.g. preparation of the intubation, application of medication on instruction) correctly. In case of threating patient harm, the nurse uses to intervene with the principles of outcome-oriented (e.g. “Perhaps you should try a laryngeal tube?“) and prohibitive (“No one of your colleagues has ever give such a dose of this medication”) speaking up [18]. The nurse always communicated without reproach and formulated clearly the sorrows, which motivated her to speak up. Each student participated in an active role in one scenario. The exact same scenarios were provided in each course.

### Collective orientation questionnaire

Collective Orientation was measured using the German version of a paper-based questionnaire. This questionnaire was developed by testing the internal and external validity of the instrument in terms of its internal structure and relationships with other variables [15]. In the German version, two items were exchanged for the subscale “dominance”. For the subscale “affiliation”, two items were added, one was removed. Within the current study, the internal consistency of the overall questionnaire (α = 0.84) as well as the subscales “affiliation” (α = 0.85) and “dominance” (α = 0.74) proved to be satisfactory. The questionnaire was applied as shown in Table 2. The 16 items were rated on a 5-point Likert scale from 1 (“totally disagree”) to 5 (“totally agree”). The questionnaire items 2, 3, 5, 6, 7, 8, 10, 11, 12, 13, 14, 15, and 16 were reversed, because the statements were phrased negatively – e.g. a 5 on the Likert scale was converted to a 1. So, a high value for “affiliation” represented the ability to work with regard for others in a team and a high value of “dominance” represented a cooperative working style as described above.

**Table 2:** Items of Collective Orientation [15]. In the German version two items were exchanged for the dominance. For the affiliation two were added and one was removed. Items marked with (R) are negatively worded and have to be reversed-scored

Collective Orientation: Subscales and Items	
Introduction: In the following you will see a series of statements. The concern is with your own option. Therefore, there are no “right” or “wrong” answers. Answer the question such that they best apply to you. Please respond to the statements in terms of your personal attitude. Items could be answered on a 5-point Likert scale from 1 (totally disagree) to 5 (totally agree).”	
German Version	English Version
Zugehörigkeit	Affiliation
Ich finde die Arbeit an Teamprojekten sehr zufriedenstellend.	I find working on team projects to be very satisfying.
Ich würde eher selbst handeln als auf den Input von anderen zu warten (R).	I would rather take action on my own than to wait around for others’
Ich bevorzuge es eine Aufgabe von Anfang bis Ende durchzuführen, ohne die Unterstützung von anderen (R).	I prefer to complete a task from beginning to end with no assistance from others.
Teams arbeiten normalerweise sehr effektiv.	Teams usually work very effectively.
Ich denke es ist normalerweise besser den Stier bei den Hörnern zu packen und etwas selber zu machen, als darauf zu warten Input von anderen zu bekommen(R).	I think it is usually better to take the bull by the horns and do something yourself, rather than wait to get input from others.
Bei den meisten Aufgaben würde ich eher allein arbeiten, als Teil einer Gruppe zu sein (R).	For most tasks, I would rather work alone than as a part of a group.
Ich kann normalerweise mehr leisten, wenn ich für mich alleine arbeite (R).	I can usually perform better when I work on my own.
Ich finde, dass es meist produktiver ist für mich alleine zu arbeiten als mit anderen (R).	I find that it is often more productive to work on my own
Ich arbeite gerne mit anderen zusammen. (only German version)	I find it easy to negotiate with others who hold a different viewpoint than I hold. (only English version)
Ich finde es nicht gut sich auf andere Teammitglieder verlassen zu müssen (only German version) (R)	I always ask for information from others before making any important decision. (only English version)
Dominanz	Dominance
Wenn ich anderen Teammitgliedern nicht zustimme, neige ich dazu meinem eigenen Bauchgefühl zu folgen (R).	When I disagree with other team members, I tend to go with my own gut feelings.
Wenn ich eine andere Meinung als ein anderes Teammitglied habe, versuche ich normalerweise bei meiner eigenen Meinung zu bleiben (R).	When I have a different opinion than another group member, I usually try to stick with my own opinion.
Es ist wichtig bei der eigenen Meinung zu bleiben, gerade wenn andere um dich herum versuchen dich zu einer Änderung zu bewegen (R).	It is important to stick on your own decision, even when others around are trying to get you to change.
Wenn andere widersprechen, ist es wichtig standzuhalten und nicht nachzugeben (R). (only German version)	When others disagree, it is important to hold one’s own ground and not give in.
Ich finde auch bei Teamarbeiten sollte man immer das tun, was man selbst für richtig hält (R). (only German version)	When solving a problem, it is very important to make your own decision and stick by it. (only English version)
Wenn ich von etwas überzeugt bin bleibe ich bei meiner Meinung, egal was andere Teammitglieder dazu sagen (R).	When others disagree, it is important to hold one’s own ground and not give in.

The questionnaire was distributed in paper form directly before and after the course and was filled out immediately by the participants. The primary outcome was the mean difference between the “Collective Orientation” immediately prior to (T1) and after the training (T2).

### Statistical analysis

Demographic survey data were analyzed in a descriptive manner and presented in mean ± standard deviation (SD). For testing hypothesis (a), the reliability of the scales was determined by Cronbach’s alpha. In order to test hypothesis (b) and (c) the Wilcoxon test was conducted. We assumed a *p* < 0.05 as being statistically significant. As an effect size, r was calculated. For all statistics, SPSS 25 (IBM Corporation, USA) was used.

## Results

Overall, the survey was completed by 147 participants. Seventy-nine persons (27 = male; 51 = female, 1 = unknown) participated in a course according to the specification “medical briefing”. Sixty-eight participants (35 = male; 31 = female, 2 = unknown) took part in a course according to the specification “TeamGAINS debriefing”. Both types of courses were conducted a total of nine times, each. The demographic data of the participants are depicted in Fig. 1.

### Reliability of the scales

In order to test hypothesis (a) Cronbach’s alpha was calculated. Subdivided by subscale and time of measurement Cronbach’s alpha was 0.78 (T1) and 0.82 (T2), respectively for the subscale “affiliation” and 0.73 (T1) and 0.78 (T2) for the subscale “dominance”. No item reduction led to a substantial increase in Cronbach’s alpha, so all items of the questionnaire were included in the following calculations.

### Pre-post-difference in collective orientation

In order to test hypothesis (b), the change of CO through the simulation training was evaluated by calculating the difference in CO before (T1) and after (T2) training. Over all participants, the “affiliation” was 3.23 ± 0.59 (*n* = 141) before and 3.13 ± 0.54 (n = 141) after the training. The difference was significant (*p* = 0.003; r = 0.25). For the subscale “dominance”, we found 3.39 ± 0.53 (*n* = 145) and 3.58 ± 0.64 (n = 145), respectively. We found the difference also being significant (*p* < 0.00; r = 0.39).

**Fig. 2:**
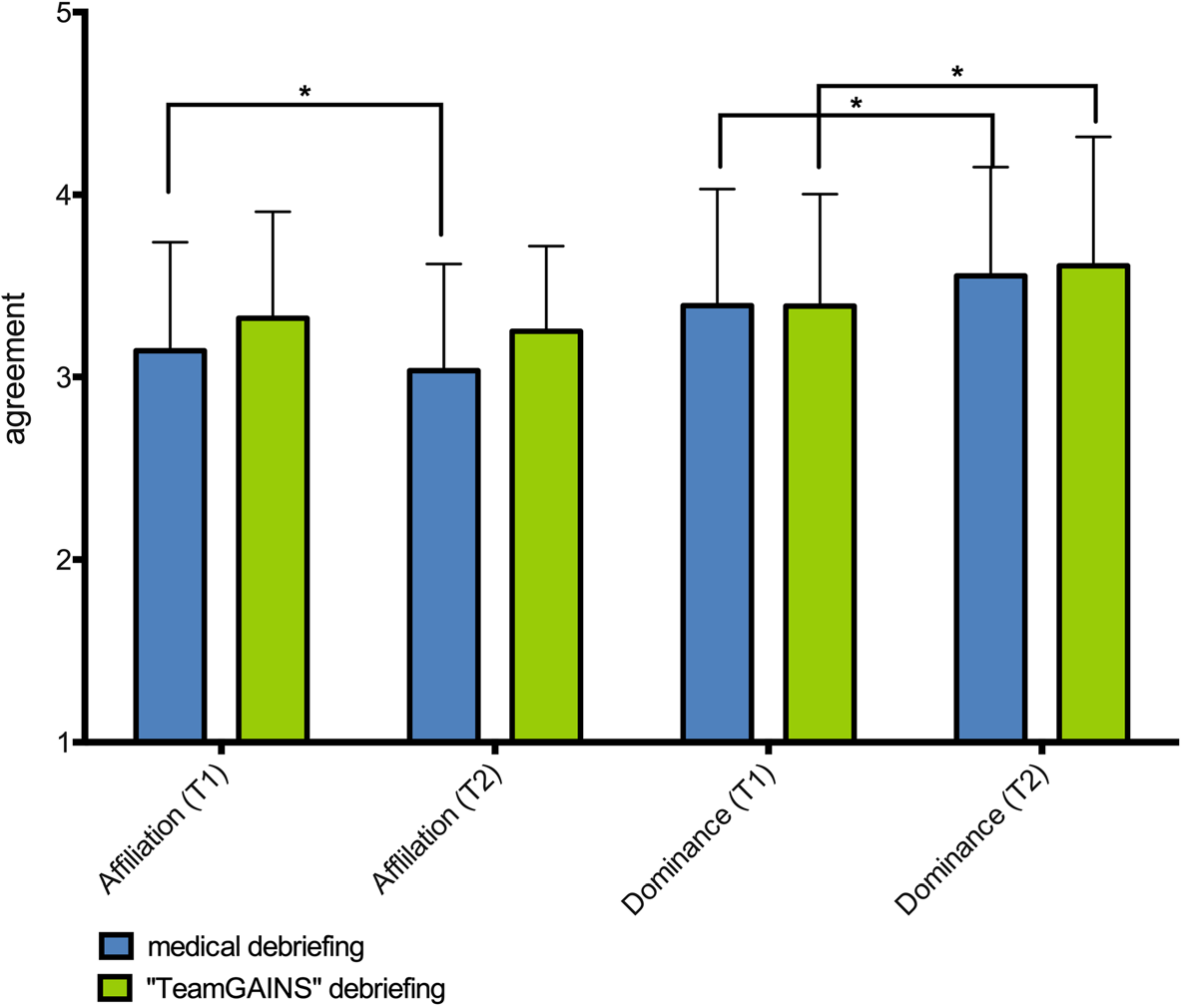
Differences in Collective Orientation prior (T1) and after training (T2). For “Medical Debriefing” "Affiliation" changes from 3.14 ± 0.6 to 3.04 ± 0.59 and “Dominance” from 3.39 ± 0.46 to 3,56 ± 0.6. For “TeamGAINS” the changes were 3.32 ± 0.58 to 3,25 ± 0.47 for “Affiliation” and 3.39 ± 0.61 to 3.60 ± 0.7 for “Dominance”

For testing hypothesis (c), we indicated a significant decline of “affiliation” in the “medical debriefing” group (3.14 (T1) to 3.04 (T2); *p* = 0.008; r = 0.31) and a significant increase in “dominance” (reverse coded) (3.39 (T1) to 3,56 (T2); *p* = 0.001; r = 0.38). In the “TeamGAINS” group, however, we could not show significant changes in “affiliation” (3.32 (T1) to 3,25 (T2); *p* = 0.148; r = 0.19), whereas the subscale “dominance” increased significantly (3.39 (T1) to 3.61 (T2); *p* = 0.001; r = 0.40). The data of the subgroups “medical debriefing” and “TeamGAINS debriefing” are shown in Fig. 2.

## Discussion

The aim of this study was to investigate whether the learning objective “role as a team member” can be integrated into an existing framework of an anaesthesia simulation course for fourth-year medical students. The intervention consists of a TeamGAINS debriefing, which included, unlike sole medical debriefing, topics of non-technical skills.

Our primary hypothesis, whether the existing scales for measuring “Collective Orientation” are applicable in our targeted group. In particular, this test was carried out as 17,2% (non-medical) students were involved of the German version of the questionnaire. All values of Cronbach’s alpha are higher than 0.7, therefore, the scales are employable without any changes and the German version of the “Collective Orientation” questionnaire can be used in our cohort.

The second aspect of the study was whether the CO can be influenced by a four-hour anaesthesia simulation training and the dependence on a debriefing. Medical students in this education phase are normally working alone, for example in exams and during their learning process. Success is often characterized by one’s own performance. Students are often focused on medical issues and do not include non-technical skills into account. Internships in Germany are carried out in clinical teams, but the success of an internship is only rated by the time of presence, not by reaching specific learning objectives.

Our findings show, that the practical training in simulation scenarios - regardless of debriefing - increases “dominance” as a surrogate for cooperative working style. Surprisingly, the effect sizes of the different debriefing types are almost the same (r = 0.4 and r = 0.38). We interpret the experiences - gained in the simulation scenarios - as responsible for this increase.

The participants are often very insecure with their anesthesiologic skills and their knowledge. In order, not to overwhelm the students and to let them successfully pass their first simulation experience, we integrated a simulated nurse into the scenarios. The nurse has the function to intervene by “speaking up” [18] in case of imminent und severe treatment errors. As a result, the nurse demonstrates an ideal team member behavior [6] in combination with a high degree of relevance for the students. Learning through observation is well described in simulation-based studies [19, 20] and can provide an explanation for the increase of “dominance” in both groups.

The value of the “affiliation” scale decreased in the investigated “medical debriefing” group but did not change in the “TeamGAINS” group. The questionnaire “CO” include questions about team members in a very generalized way. In our scenario two students and the simulated nurse were working in a team in each scenario. Due to the fact that the nurse was an instructor, the student perhaps did not assess this position as being a team member. The structure of the debriefing supported this view, as the nurse war absent during the debriefing.

The collaboration of two students in a team, did lead to a decreased “affiliation”. In our interpretation, the students in the “medical debriefing” group rated the overall performance as their sole responsibility. This interpretation would match all examination formats during their medical training, in which the students work alone and are examined individually. The team partner “student” with a comparable level of knowledge might not be considered as a profitable partner. The TeamGAINS debriefing with contents of the non-technical skills framework migrated these effects and demonstrates potentials to actively use other team members with the same level of knowledge (e.g. task division, arguing of different perspectives). Perhaps “affiliation” did not increase in this group because students are not yet able to estimate the potential benefit for the clinic setting. Maybe a one-time intervention (simulation based training) is not enough for an effect.

Kolbe et al. showed, that TeamGAINS increased psychological safety and leader inclusiveness in trainees of physicians and nurses. In particular, step four of the TeamGAINS debriefing deals with the perspectives of the team member (circular question, advocacy-inquiry) and values them for the treatment process [10]. We interpret that creating the transparency of the team’s view is responsible for the higher values of “affiliation”, comparable to the increase of leader inclusiveness. These results show how complex learning in a simulated environment is. Further research is needed to explore the impact of details in a scenario to improve medical students learning experience.

According to our results, medical students have the opportunity to change their attitudes towards teamwork, despite the load of learning medical content [21, 22]. In another study with a 90-min non-technical skills intervention in a collective of fourth year medical students, teamwork behavior could be significantly improved [23]. In this study, an intervention (90 min) for non-technical skills was performed and their influence checked in emergency medicine scenarios. Nicolaides et al. found a plethora of feasible intervention in the literature to improve non-technical skills, and recommended the outcome parameters knowledge, skill performance and attitude towards skills [24].

Due to the briefness of simulation courses for medical students and their restricted availability, we see Nicolaides’s recommendation as a requirement for an obligatory longitudinal non-technical skills curriculum.

Our findings indicate that it is hardly possible not to interfere non-technical skills by a simulation-based team training. For this reason, the topics should be included into a debriefing on a regular basis.

### Limitations

Studying medicine in Germany is often dependent on local circumstances. At the place of this study, a model-concept of studying medicine is currently carried out. During the study phase (fourth year), the medical students can freely organize various internships in unrestricted selectable medical specialties. So, previous experiences and expectations regarding the importance of teamwork are different between the individual students. Changes in CO may be depended on characteristics of the participants and characteristics and performance of the students´ team partner. We tried to minimize these influences by including a great number of participants. We did not rate the students’ performance according to the ANTS framework and therefore could not correlate “Collective Orientation” with the ANTS objectives. Furthermore, we did not assess, how long any training effect might persist.

## Conclusion

The “Collective Orientation” questionnaire can be applied to fourth year medical students. Anaesthesia simulation courses have an influence on the attitude towards teamwork. The influence is negatively to the subscale “affiliation” by a “medical debriefing” and independently regardless of the nature of the debriefing for the subscale “dominance”.

Simulated team members have the potential to carry out a positive influence by being a role model. We recommend a debriefing for medical students using the TeamGAINS approach to clarify the connection between the individual performance and non-technical skills - otherwise negative effects in “affiliation” may occur.

Anaesthesia simulation courses have the potential being a part of a longitudinal education curriculum for teaching non-technical skills. Further research has to show which components of a simulation courses can improve student’s behavior.

## Data Availability

The datasets used and/or analyses during the current study are available from the corresponding author on reasonable request.

## Abbreviations

ANTS: Anaesthesists-Non-Technical Skills
CO: Collective Orientation
NTS: non-technical skills
TeamGAINS: Guided team self-correction, advocacy-inquiry and systemic-constructivist techniques
